# Childhood maltreatment increases the suicidal risk in Chinese schizophrenia patients

**DOI:** 10.3389/fpsyt.2022.927540

**Published:** 2022-09-20

**Authors:** Peng Cheng, Peijun Ju, Qingrong Xia, Yuanyuan Chen, Jingwei Li, Jianliang Gao, Loufeng Zhang, Fanfan Yan, Xialong Cheng, Wenzhi Pei, Long Chen, Cuizhen Zhu, Xulai Zhang

**Affiliations:** ^1^Department of Science and Education, Affiliated Psychological Hospital of Anhui Medical University, Hefei, China; ^2^Anhui Clinical Center for Mental and Psychological Diseases, Hefei Fourth People's Hospital, Hefei, China; ^3^Anhui Clinical Research Center for Mental Disorders, Anhui Mental Health Center, Hefei, China; ^4^Shanghai Key Laboratory of Psychotic Disorders, Shanghai Mental Health Center, Shanghai Jiao Tong University School of Medicine, Shanghai, China; ^5^Shanghai Key Laboratory of Psychotic Disorders, Shanghai, China

**Keywords:** suicide risk, suicide ideation, suicide behavior, schizophrenia, child trauma

## Abstract

**Objectives:**

Childhood trauma might be a modifiable risk factor among adults with serious mental illness. However, the correlation of child trauma and suicide is unclear, which were cited most frequently as the biggest challenge to schizophrenia (SCZ) patients in China. We aim to study relationships between child trauma and suicide in SCZ patients of different disease stages.

**Methods:**

Ninety-one participants were included and divided into two groups, namely, first-episode group (*n* = 46), relapsed group (*n* = 45). The Positive and Negative Syndrome Scale was used to evaluate the severity of psychotic symptoms. The Beck's Suicide Intent Scale and The Nurses' Global Assessment of Suicide Risk were conducted by patient self-report to assess suicide symptom. The childhood trauma questionnaire was used to estimate severity of traumatic stress experienced during childhood.

**Results:**

Childhood trauma and different dimensions of suicide were significantly higher in the relapsed group than first-episode group (*P* < 0.01, respectively). BMI has a significant positive relationship with recent psychosocial stress (*β* = 0.473, *t* = 3.521, *P* < 0.001) in first-episode group. As in relapsed group, BMI has a positive effect between severe mental illness and suicide ideation (*β* = 0.672, *t* = 5.949, *P* < 0.001; *β* = 0.909, *t* = 2.463, *P* < 0.001), Furthermore, emotional neglect presented positively related to the suicide risk and proneness to suicidal behavior (*β* = 0.618, *t* = 5.518, *P* < 0.001; *β* = 0.809, *t* = 5.356, *P* < 0.001).

**Conclusion:**

Relapsed group of patients had significantly more severe childhood trauma, recent psychosocial stress, suicidal risk and proneness to suicidal behavior. BMI and emotional neglect are unique predictors for different dimensions of suicide.

## Introduction

Schizophrenia (SCZ) is a complex dysfunction of genetic, heterogeneous behavioral and cognitive syndrome that is prevalent in ~1% of the general population around the world, whose origins etiology appear to lie in genetic and/or environmental disruption of brain development (1). Life expectancy for people with SCZ is probably 10–25 years shorter than that for the general people (2, 3). It has long been recognized that individuals with traumatic experiences among persons are at increased risk of subsequently developing poor outcomes, including substance abuse (4), cognitive deficits (5), sexual dysfunction (6), personality disorders (7) and dissociative disorders (8). In recent years, abundant evidence has identified that traumatic events play a causal role in serious mental illnesses, including depression (8), anxiety disorders (9) and eating disorders (10), as well as SCZ (11), these findings has accumulated revealing that trauma/stressful events in childhood/adolescence have potential etiological relationship between the psychotic disorders (12), and these child traumatic events as a socio-environmental risk factor promote the psychotic symptoms evolving (13). A recent review and meta-analysis recommended that childhood traumatic events had relationship with offending behavior in SCZ. A dose-response-relationship was assumed that the treatment outcomes to be poorer in SCZ when there was a history of childhood maltreatment. Therefore, more efforts should be devoted on this research aspect (14).

Over recent decades, studies have consistently documented markedly elevated worldwide mortality in mental illnesses, notably, suicide accounted for major reason of unnatural deaths (15) especially in persons with SCZ (16). Sukanta Saha explored that the risk of suicide was approximately 13 times higher for persons with SCZ compared with the general population (17). It has been proposed among SCZ patients that the lifelong risk of suicide is around 5% (18), the rate of population-attributable risk of suicide is 8.9% (19), the rate of suicide attempt is 25–50% (20), and the rate of suicidal ideation at least once is 43–79% (21). It has been estimated up to 50% of those suffering from SCZ might experience suicidal ideation, with or without suicidal attempt, at some time during the course of the illness, usually 4.9% commit suicide from first admission or near illness onset (18). As well as, studies have also observed that 38% had at least 1 episode of self-harm in a 2–12-year follow-up period (22, 23). Furthermor, recurrent relapses, impairments of societal and occupational functioning, severity of the disease and realistic understanding of the harmful influence of disorder are regarded as schizophrenia-specific suicide risk factors (23). The higher risk of suicide among SCZ patients reveals that it is necessary to evaluate the disease-related factors and sociocultural factors for the improved identification of risky individuals and development of preventive interventions. It is worth noting the fact that most patients with SCZ experience relapses, and only less than one quarter of patients achieved complete recovery in long-term follow-up study. Hence, the different stages of disease is related to heterogenous psychotic symptoms of patients, with potentially distinct risk to suicide, Therefore, it is crucial to understand the different pattern of disease and its possible predictors for suicidal risks in this vulnerable population (24).

The existing studies have tried to identified several factors predicting the risk of suicide in individuals with SCZ, which include younger age, male gender, lack of social support, unemployment, being unmarried, being well-educated, being intelligent (21), a family history of psychiatric disorder, having poor work functioning, higher number of hospitalizations (25), presence of a previous suicide attempt, poor adherence to treatment, history of suicidal behavior (20), having had recent (i.e., within past 3 months) life events (26), having access to lethal means, and seriousness of psychiatric pathology (21), comorbid affective disorder and the strong stigma of mental illness in rural areas (16). Furthermore, some studies have also reported that a combination of genetic markers and early traumatic life events might indeed be used to identify patients at risk for suicide attempt in SCZ. Nevertheless, unique patterns of the early stressful events seem difficult to say that these factors are useful in predicting and preventing suicide in the SCZ, moreover, the existing researches are predominantly conducted in the Western countries (e.g., the USA, UK and Sweden) and many trends may not be generalizable to non-Western countries, thus the predictors for the suicide of persons with SCZ in China are unknown.

There are few studies that investigates the relationship between childhood maltreatment and their potentially distinct effects on suicide of SCZ patients in China. We hypothesized that childhood trauma events such as abuse, physical neglect and emotional neglect play vital effect during the onset or relapse course of the illness in the patients suffering from SCZ. In light of this information, we aimed to evaluated the relationship between early stress life events and clinical features of SCZ, and explore the risk factors which contribute to increasing knowledge relating to suicidal suicide attitude and suicide attempts behavior in SCZ patients.

## Materials and methods

### Ethics statement

Medical Ethics Committee of the Anhui Mental Health Center (AMHC) approved this study, all participants provided written consent prior to study participation in accordance with the principles of the Declaration of Helsinki. The trial clinical registration number was ChiCTR2100045240.

### Procedure

**Figure 1:**
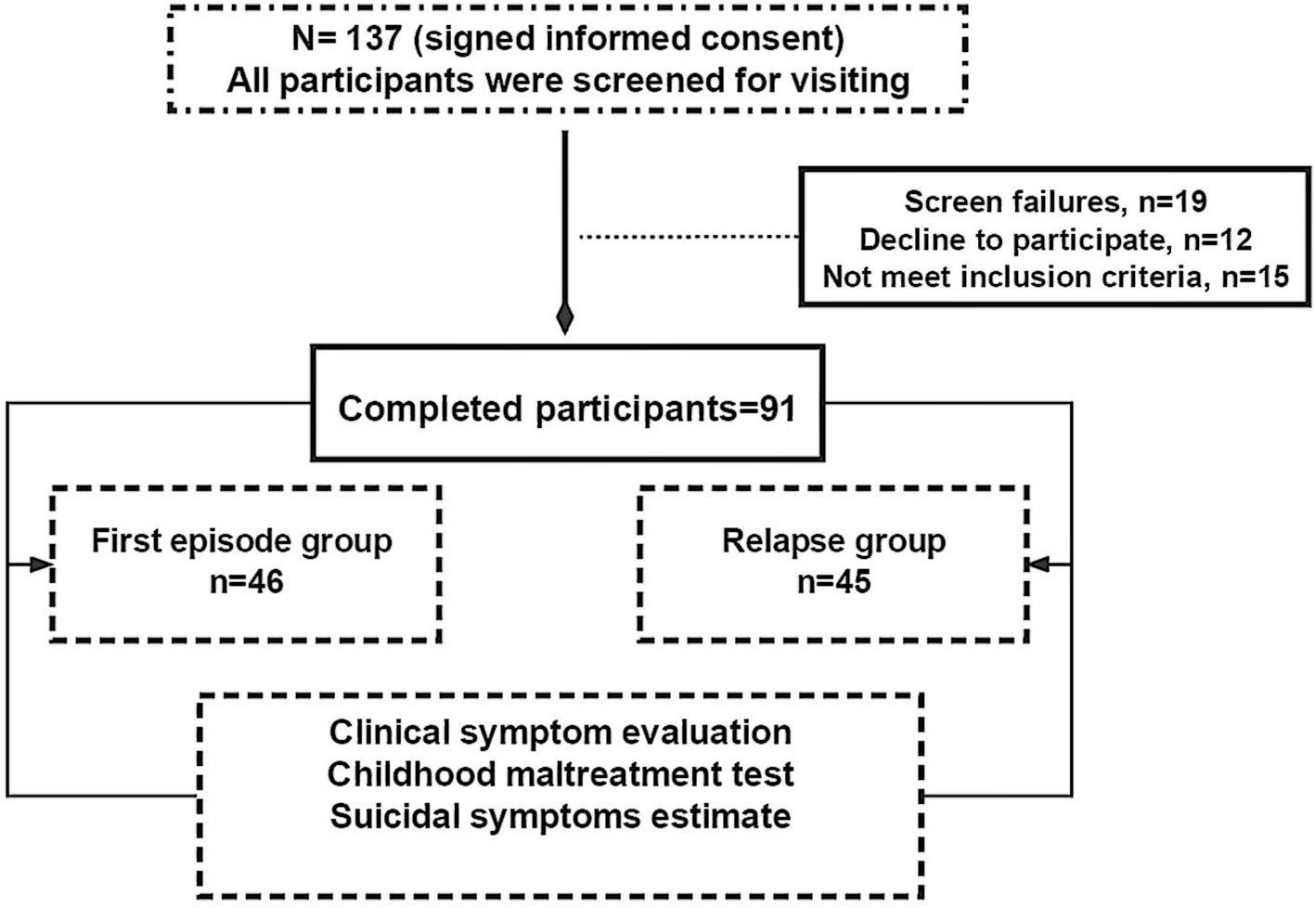
Study procedure.

This pilot study explored the relationship about the childhood traumatic stress related suicidal symptoms in SCZ patients. A total of 137 participants were initially selected. Of these, 15 participants did not meet the inclusion criteria, 19 individuals could not complete the clinical estimate, and 12 individuals refused to sing informed consent. Hence, total of 45 subjects who did not meet the experimental criteria were excluded from present study. Ultimately, the remaining 91 participants were included in this study and divided them into two groups according to the Diagnostic and Statistical Manual of Mental Disorders, Fifth Edition (DSM-5) (27)–first episode group (SCZ patients of first episode, *n* = 46), relapsed group (relapsed SCZ patients, *n* = 45). All patients were hospitalized at the AMHC between February 2018 and August 2021 (Figure 1).

All participants were assessed by the two professional psychiatrists through the Mini-International Neuropsychiatric Interview (MINI) 6.0.0. According to the trial standards, patients of first episode group and relapsed group diagnosed with SCZ met the criteria of the DSM-5. The inclusion criteria for the patients in the first episode group were as follows: ① age = 18–60 years, ② fulfillment of the DSM-5 criteria for SCZ, ③ first-episode state SCZ. The inclusion criteria for the patients in the recurrent patient group were as follows: ① fulfillment of the first and second third criteria of first episode group. ② patients was treated with second generation anti-psychotic drugs, drug treatment matching between the two groups. The exclusion criteria were as follows: ① people with serious physical diseases and substance abuse, ② a history of craniocerebral trauma, organic cerebral diseases, or other mental disorders, ③ had received electroconvulsive therapy or transcranial magnetic stimulation within 6 months, ④ pregnant or lactating women. All subjects received two trained professional psychiatrists to assess basic sociodemographic data, clinical symptom.

### Clinical assessments

#### Mini-international neuropsychiatric interview 6.0.0

The preliminary clinical diagnosis was verified by MINI 6.0.0. It is a concise diagnostic interview for psychiatric disorders exploited jointly by psychiatrists in the United States and Europe. All patients underwent the MINI 6.0.0 to confirm the clinical diagnosis SCZ (28).

#### The positive and negative syndrome scale

PANSS was used worldwide to estimate psychiatric symptoms for decades. Versions of the five-factor model have been used in diverse SCZ research areas including positive symptom, negative symptom, cognitive defect, hostility/excitement symptom and anxiety/depression symptom (26).

#### The beck's suicide intent scale

BSIS is one of the most commonly used clinician-rated measurements of suicidal symptoms in studies. It has 19 items and is divided into suicidal ideation and suicidal tendency. The higher scores are, the higher levels of suicidal ideation and suicidal tendency is (29).

#### The nurses' global assessment of suicide risk

NGASR is one of the most commonly used clinician-rated measurements of suicidal symptoms in studies as well (30). It has been proven to have good reliability and validity, with a Cronbach's alpha coefficient of 0.88 and an intra-class coefficient of 0.9 (30, 31). The higher scores are, the more severe the symptoms are.

#### The childhood trauma questionnaire

CTQ is a widely used instrument for measuring the severity of traumatic stress experienced during their childhood. It has been utilized for the assessment of emotional abuse, physical abuse, abuse, sexual abuse, emotional neglect, physical neglect, as shown in Table 2. A Mandarin version of a five-subscale model of the CTQ has been proven to have good reliability and validity (32).

## Statistical analysis

The *t*-tests and chi-squared test were performed to compare the differences in either continuous or categorical parameters between the first episode patient group and relapsed patient group. We used spearman correlation coefficients with false discovery rate (FDR) judgment in each group to constructed the relationship between possible correlation factors and scales of PANSS, NGASR, BSIS and CTQ. Stepwise linear regression analysis was used to conducted the relationship and interaction among predicted risk factors, childhood trauma and different dimensions of suicide in the first-episode group and relapse group. All statistical tests were two-tailed tests, and statistical significance was set as α < 0.05. All analyses were conducted using the SPSS version 22.0 (IBM Corp).

## Results

### Results of demographic background, psychotic symptom, suicidal symptom and childhood trauma between in the two groups

Overall, there was no significant difference in demographic background such as age, gender, BMI and years of education between two groups (*P* ≥ 0.05). Moreover, we also found there was no significant difference in clinical symptoms between the first-episode group and relapse group, either in the total scores of the PANSS scale or in the subscale scores of positive symptoms, subscale scores of negative symptoms, subscale scores of hostility/excitement symptoms, subscale scores of anxiety/depression symptoms and subscale scores of disorganized thoughts (*P* ≥ 0.05). Results was shown in Table 1. Furthermore, we investigated the occurrence of childhood trauma, suicide risk, suicide ideation and suicide behavior between the first-episode group and recurrence group, the results showed that the patients of recurrence group had more severe suicide risk than patients of first episode group (*p* = 0.02). Importantly, the results revealed the patients of recurrence group had more severe mental illness, more severe recent psychosocial stress and more likely proneness to suicidal behavior than the patients of first episode group (*P* < 0.01, respectively). Meanwhile, we also found that the level of childhood trauma in the relapse group was significantly serious than that in the first episode group. That is to say, they were more likely to face emotional abuse, emotional neglect and physical neglect (*P* < 0.01, respectively). Results was shown in Table 2.

**Table 1:** Demographic information and psychotic symptom, suicidal symptom of participants.

**Items and subscales**	**FEP(*n* = 46)**	**RP(*n* = 45)**	***t***	***P***
Age	28.39 ± 9.10	31.00 ± 7.78	−1.47	0.15
BMI	22.99 ± 3.64	21.73 ± 3.50	1.68	0.10
Male (percentage%)	23(50.00%)	21(51.20%)	−1.15	0.25
Years of education	9.93 ± 4.46	11.24 ± 3.73	−1.52	0.13
**PANSS**				
Positive symptoms	22.59 ± 6.24	21.84 ± 5.86	0.59	0.56
Negative symptoms	17.89 ± 5.85	19.00 ± 6.05	−0.89	0.38
Hostility/excitement symptoms	9.54 ± 4.00	9.47 ± 3.53	0.97	0.92
Anxiety/depression symptoms	10.74 ± 3.42	11.67 ± 3.52	−1.28	0.21
Disorganized thoughts	7.78 ± 2.49	8.04 ± 2.02	−0.55	0.58
Total score	79.90 ± 18.60	80.00 ± 17.42	−0.29	0.77

FEP, first episode patient; RP, relapsed patient; BMI, body mass index; PANSS, positive and negative syndrome scale; P < 0.05: statistically significant.

**Table 2:** Comparisons in CTQ, suicidal symptoms and psychotic symptoms.

**Scale**	**FEP(*n* = 46)**	**RP(*n* = 45)**	***t***	***p***
**BSIS**				
Suicide risk	12.46 ± 4.10	24.07 ± 23.65	154.75	0.02
Suicide ideation	14.35 ± 14.11	12.62 ± 3.51	451.44	0.43
**NGASR**				
Suicide mood	1.98 ± 1.02	1.60 ± 1.39	12.50	0.142
Severe mental illness	0.74 ± 0.65	1.62 ± 1.03	10.72	<0.01
Proneness to suicidal behavior	0.80 ± 0.72	1.51 ± 1.44	24.32	<0.01
Recent psychosocial stress	0.33 ± 0.56	0.67 ± 0.48	0.22	<0.01
**CTQ**				
C1(emotional abuse)	7.57 ± 1.80	11.71 ± 3.58	12.98	<0.01
C2 (physical abuse)	9.17 ± 3.36	9.13 ± 4.43	1.32	0.96
C3 (sexual abuse)	6.65 ± 2.88	7.96 ± 3.77	4.42	0.68
C4(emotional neglect)	8.15 ± 2.35	10.98 ± 4.47	15.77	<0.01
C5(physical neglect)	7.78 ± 2.16	10.38 ± 3.39	9.58	<0.01

FEP, first episode patient; RP, relapsed patient; BSIS, Beck's suicidal ideation scale; NGASR, The Nurses' Global Assessment of Suicide Risk scale; CTQ, childhood trauma questionnaire. P < 0.05: statistically significant.

### The correlation between childhood trauma and suicide risk, suicide ideation and suicidal behavior

Tables 3, 4 presents the results of the spearman correlation analysis to compare the correlation about the child trauma and different dimensions of proneness to suicidal behavior in the first-episode group and relapse group. The results shown physical abuse and sexual abuse had positive related to recent psychosocial stress in the patients of first-episode group (*r* = 0.312, *P* < 0.05; *r* = 0.334, *P* < 0.05). Meanwhile, we found sexual abuse had the positive related to suicidal ideation (*r* = 0.428, *P* < 0.001), especially, the findings showed that emotional neglect had positive correlation to recent psychosocial stress, severe mental illness, suicide risk, suicidal ideation and proneness to suicidal behavior (*r* = 0.451, *P* < 0.001; *r* = 0.657, *P* < 0.001; *r* = 0.681, *P* < 0.001; *r* = 0.848, *P* < 0.001; *r* = 0.566, *P* < 0.001; respectively) in the patients of relapse group. Interestingly, we found index of BMI appeared the positive interrelated to the recent psychosocial stress, suicide risk, suicidal ideation and proneness to suicidal behavior (*r* = 0.473, *P* < 0.001; *r* = 0.597, *P* < 0.001; *r* = 0.791, *P* < 0.001; *r* = 0.524, *P* < 0.001; respectively) in the patients of relapse group. As well as, the results revealed the years of education had positive interrelated to mental illness (*r* = 0.672, *P* < 0.001) in the patients of relapse group. Moreover, results explored only suicide risk had positive relationship with the psychotic symptom in the patients with first-episode (*r* = 0.331, *P* = 0.035), while, both of suicide risk, suicidal ideation, suicide mood, severe mental illness, proneness to suicidal behavior and recent psychosocial stress have positive correlation (*r* = 0.390, *P* = 0.0008; *r* = 0.434, *P* = 0.0003; *r* = 0.545, *P* < 0.001; *r* = 0.540, *P* < 0.001; *r* = 0.358, *P* = 0.0016; *r* = 0.380, *P* = 0.010) with psychotic symptom in the replace group, especially, the emotional neglect also presented the positive relation to the psychotic symptom in the replace group, these results may be seen in the Table 5.

**Table 3:** Correlation between demographic and childhood trauma risks with suicide factors in the relapse group.

**Factors**	**Suicide risk**		**Suicidal ideation**		**Recent psychosocial stress**		**Severe mental illness**		**proneness to suicidal behavior**		**Suicide mood**	
	***r***	***p***	***r***	***p***	***r***	***p***	***r***	***p***	***r***	***p***	***r***	***p***
Gender	−0.133	0.385	−0.039	0.802	−0.127	0.405	0.161	0.618	0.013	0.935	0.012	0.935
Age	−0.156	0.271	0.171	0.891	0.184	0.218	0.618	0.837	0.144	0.253	0.191	0.209
BMI	0.597^**^	<0.001	0.791^**^	<0.001	0.473^**^	<0.001	0.057	<0.001	0.524^**^	<0.001	−0.034	0.826
YE	0.019	0.903	0.190	0.188	−0.043	0.719	0.672^**^	0.001	0.175	0.411	0.930	0.290
**CTQ**												
C1	0.217	0.290	0.029	0.435	−0.098	0.466	−0.067	0.561	−0.217	0.323	0.088	0.567
C2	0.032	0.462	0.183	0.069	0.043	0.990	0.136	0.189	0.064	0.260	0.338	0.239
C3	0.242	0.159	0.428^**^	<0.001	0.029	0.912	0.230	0.305	0.075	0.444	0.402	0.069
C4	0.618^**^	<0.001	0.848^**^	<0.001	0.451^**^	<0.001	0.657^**^	<0.001	0.566^**^	<0.001	0.931	0.055
C5	0.124	0.541	0.231	0.244	0.052	0.042	0.075	0.635	−0.120	0.435	0.204	0.189

Correlation between demographic and childhood trauma risks with suicide factors in the relapse group. RP, relapsed patient; CTQ, childhood trauma questionnaire, C1 (emotional abuse), C2 (physical abuse), C3 (sexual abuse), C4 (emotional neglect), C5 (physical neglect). The false discovery rate (FDR) method is used to adjust the p-value.

* p ≤ 0.05;

** p ≤ 0.001.

**Table 4:** Correlation between demographic and childhood trauma risks with suicide factors in the first episode group.

**Factors**	**Suicide risk**		**Suicidal ideation**		**Recent psychosocial stress**		**Severe mental illness**		**Proneness to suicidal behavior**		**Suicide mood**	
	***r***	***p***	***r***	***p***	***r***	***p***	***r***	***p***	***r***	***p***	***r***	***p***
Gender	−0.020	0.894	−0.137	0.362	0.008	0.956	−0.183	0.223	−0.050	0.742	0.009	0.953
Age	0.226	0.131	0.193	0.198	0.031	0.841	0.230	0.124	−0.076	0.618	−0.052	0.731
BMI	0.002	0.990	0.116	0.443	−0.206	0.108	−0.108	0.474	0.042	0.779	−0.236	0.114
YE	0.195	0.195	0.060	0.691	−0.009	0.892	0.024	0.875	−0.005	0.972	0.029	0.847
**CTQ**												
C1	0.044	0.770	−0.040	0.791	0.188	0.707	0.009	0.951	−0.155	0.303	−0.099	0.511
C2	0.105	0.488	0.124	0.410	0.312^*^	0.007	0.188	0.211	0.065	0.666	0.017	0.908
C3	−0.020	0.893	−0.062	0.684	0.334^*^	0.008	0.114	0.451	0.193	0.199	0.033	0.826
C4	0.014	0.926	0.028	0.853	0.232	0.327	0.198	0.188	0.141	0.349	0.061	0.688
C5	0.081	0.592	0.038	0.805	0.262	0.136	0.192	0.201	0.157	0.297	0.190	0.205

RP, relapsed patient; CTQ, childhood trauma questionnaire, C1 (emotional abuse), C2(physical abuse), C3 (sexual abuse), C4(emotional neglect), C5 (physical neglect). The false discovery rate (FDR) method is used to adjust the p-value.

* P ≤ 0.05;

** P ≤ 0.001.

**Table 5:** Correlation analysis between PANSS about suicide-related risks and childhood trauma.

**Factors**	**PANSS total score**			
	**FEPs**		**RPs**	
	***r***	***p***	***r***	***p***
Suicide risk	0.311^*^	0.035	−0.390^**^	0.008
Suicidal ideation	0.047	0.758	−0.434^**^	0.003
Suicide mood	−0.081	0.592	−0.545^**^	<0.001
Severe mental illness	−0.060	0.691	−0.540^**^	<0.001
Proneness to suicidal behavior	0.225	0.133	−0.358^*^	0.016
Recent psychosocial stress	0.054	0.721	−0.380^*^	0.010
**CTQ**				
C1	−0.028	0.856	−0.232	0.125
C2	−0.174	0.247	0.047	0.760
C3	−0.020	0.895	0.056	0.714
C4	0.052	0.733	−0.464^**^	0.001
C5	0.076	0.616	−0.243	0.108

Correlation analysis between PANSS about suicide-related risks and childhood trauma. FRP_S_, first episode group; RP, relapsed patient; PANSS, positive and negative syndrome scale; CTQ, childhood trauma questionnaire, C1 (emotional abuse), C2 (physical abuse), C3 (sexual abuse), C4 (emotional neglect), C5 (physical neglect). The false discovery rate (FDR) method is used to adjust the p-value.

* p < 0.05;

** p < 0.001.

### Prediction risk factors of child trauma for recent psychosocial stress and suicide dimensions

Stepwise regression analysis of sociodemographic characteristics and child trauma factors were performed to predict recent psychosocial stress and different dimensions of suicide. In the first episode group of the final model from forward regression indicated that the index of BMI accounted for 22.4% of the variance recent psychosocial stress, A significant positive relationship was found between index of BMI and recent psychosocial stress (*β* = 0.473, *t* = 3.521, *P* < 0.001). As in the relapse group, the final model from forward regression indicated that the index of BMI accounted for 45.1% of the variance severe mental illness, meanwhile, emotional neglect of accounted for 45.1 and 41.4% of the variance about suicide risk and proneness to suicidal behavior, respectively. We found the variables of BMI and emotional neglect accounted for 75.5% of the variance in suicide ideation. A positive effect was explored between BMI and severe mental illness (*β* = 0.672, *t* = 5.949, *P* < 0.001). Furthermore, emotional neglect presented the positive related to the suicide risk and proneness to suicidal behavior (*β* = 0.618, *t* = 5.518, *P* < 0.001; *β* = 0.809, *t* = 5.356, *P* < 0.001), in addition, index of BMI and emotional neglect had positive correlation to the suicide ideation (*β* = 0.909, *t* = 2.463, *P* = 0.018), these results indicate that the index of BMI and emotional neglect in prone to be a predictive risk factor for the recent psychosocial stress and suicide risk, suicide ideation and suicide behavior. Results was shown in Table 6.

**Table 6:** Prediction of risk factors for the different dimension of suicide.

**Groups**	**dependent variables**	**independent variables**	**B**	**SE**	**Beta (*β*)**	***t***	***P***	***R^2^***
FEPs	Recent psychosocial stress	BMI	0.064	0.018	0.473	3.521	<0.001	0.224
RPs	Severe mental illness	BMI	0.198	0.033	0.672	5.949	<0.001	0.451
	Suicide risk	C4	3.268	0.634	0.618	5.158	<0.001	0.382
	proneness to suicidal behavior	C4	0.260	0.049	0.809	5.356	<0.001	0.414
	Suicide ideation	BMI	0.911	0.370	0.909	2.463	0.018	0.755
		C4	1.361	0.289	1.737	4.709	<0.001	

Gender, age, BMI, years of education and factors of childhood maltreatment were included in stepwise regression analysis to predict the risk factors of suicide between the two groups. FEP, first episode patient; RP, recurrent patient; BMI, body mass index. C4 (emotional neglect). Significant level at p < 0.05. B, unstandardized coefficient; SE, Standard error, Beta: standardized coefficient; R^2^, R square.

## Discussion

The results of this study are summarized as follows: firstly, compared to the patients of first episode group, patients of relapse group had significantly more severe child trauma, recent psychosocial stress, suicidal risk and proneness to suicidal behavior. Secondly, physical abuse and sexual abuse had positive related to recent psychosocial stress in the patients of first-episode group. Meanwhile, in the patients of relapse group, sexual abuse had the positive related to suicidal ideation, especially, emotional neglect had positive correlation to recent psychosocial stress, severe mental illness, suicide risk, suicidal ideation and proneness to suicidal behavior. Moreover, index of BMI appeared the positive interrelated to the recent psychosocial stress, suicide risk, suicidal ideation and proneness to suicidal behavior. Thirdly, index of BMI was a unique predictor of severe mental illness and suicidal ideation. Fourthly, emotional neglect was a unique predictor of suicidal risk, suicidal ideation and proneness to suicidal behavior, whereas results shown no unique risk factor for the different dimensions of suicide in the patients of first episode group, suggesting that child emotional neglect are unique predictor for the relapsed SCZ patients.

In the present study, patients of relapsed group had higher current of child trauma and severe mental illness than those patients of first episode. According to the World health Organization (WHO), each year, about one million people die by suicide across the world. Scholars also found that all ages are at risk for suicide. Suicidal ideation and behavior are relatively common in healthy, It is worth noting that more than 90% of victims of suicide have a psychiatric disorder (23), in addition, a history of child trauma may lead to increased risk for psychiatric symptoms (29) and suicidal attempt in the SCZ (33). However, more recent research explored the heterogeneity in the relationship between child trauma and psychotic symptoms, a study of large population-based sample discovered intention to harm is the key factor linking child trauma and general psychopathology symptoms of hallucinations and delusions, rather than specific psychotic symptoms in isolation, these finding are consistent with present study, furthermore, no evidence supported that the specific type of child trauma had associations between with particular psychotic symptoms (30). Meanwhile, our finding observed that the patients of relapsed group had higher current of recent psychosocial stress, suicide risk, and proneness to suicidal behavior than those patients of first episode. Evidence of risk factors related to symptomatology shown that in an early part of the course of illness and not using antipsychotic drugs are associated with greater suicide risk in patients with SCZ (30), uniquely, excess mortality is seen mostly in patients lack of adherence to antipsychotic drugs treatment (34), specifically, recurrent relapses are regarded as SCZ-unique suicide risk factors (23), these outcome are unanimous with the results of present study, these results reminded us that recurrent relapses may contribute to limited suicidal ideation quickly intensify to a suicide attempt during early stages of SCZ.

There is abundant evidence indicating that anomalous psychosocial characteristics were consistently associated with elevated risk of suicide, and chronic psychosocial stress may be a potential predictor for suicide (35). Weight gain in SCZ patients due to the use of antipsychotic drugs is a leading cause of non-compliance, which is risk leading to increased relapse (36). Moreover, overweight is also linked to greater morbidity, mortality and lower quality of life, being obese can affect psychological wellbeing and form chronic psychological stress (35). Earlier studies investigated the association of BMI with suicidality among adolescents, they found that adolescent overweight increased the risk for suicide ideation up to 1.30 fold, interestingly, the author found if adolescents' perceived weight was entered into the analyses, the risk for suicide ideation became non-significant, these results suggest that perceived weight may be a mediator in the association of BMI with proneness to suicidal behavior (37). Although there is some circumstantial evidence for weight gain as a psychosocial stress, in view of the paucity of adults studies means, it is not possible to draw a clear conclusion on role of obese in the being risk indices for suicide (35). In the present study, the increase of BMI index was not only related to psychological stress, but also closely related to suicide risk, suicidal ideation and proneness to suicidal behavior in the patients of relapsed group. These results reminded us the obese may be the predictive risk factor for the development of psychosocial stress and that we should take into account an action on the suicidal impulsive behavior in advance.

It is well-documented that individuals experienced the physical abuse, sexual abuse and emotional neglect of childhood adversities, have been related to the development of adult onset psychosis and SCZ (38). Specifically, SCZ patients who have a history of physical and/or sexual abuse have also been found the increased rates of hallucinations, delusions, or thought disorder (38), as well as the number and severity of childhood maltreatment may elevate symptoms of hallucinations and delusions, such findings highlight childhood maltreatment could be as meaningful factors in the development of later psychotic symptomatology (39). Which is similar to findings of this present study shown that the physical abuse and sexual abuse had positive correlation with chronic psychosocial stress of first episode group and suicide ideation of relapsed group. Although there are some mixed discoveries in these contexts, such Laura P et al. in his systematic review and meta-analysis explored no statistically significant correlation between a history of sexual abuse and a lifetime diagnosis of SCZ, but the author demonstrated the association between sexual abuse and psychiatric disorders persisted regardless of sex of the abuse survivor or age at which abuse occurred (40). In addition, in the nearly, evidence of scientific highlighted the early traumatic life events may have a substantial impact on essential brain structure and functions, which may persist throughout adulthood (41), the interaction between the brain and the external environment can be mediated by epigenetic alterations in gene expression, the specific molecular mechanisms leaves long-lasting marks that cause subsequent pathology and physiology. On the other hand, it is well-known that the lack of exposure to adequate stimuli (as is the case with emotional neglect) can also have profound effects on neurodevelopment (42). Typically, these finding might be similar with our results that effects of emotional neglect had positive related to the chronic psychosocial stress, severe mental illness, suicide risk, suicidal ideation and proneness to suicidal behavior, moreover, emotional neglect is expected as a more predictive risk factor, linking maltreatment exposure to suicide in the patients of relapsed group. It is essential comprehensive to understand these vulnerability risk factors to suicide as a promising future treatment and prevention measures in the field.

### Limitations

Despite we gain some insight between the child trauma and suicide through studies discussed above, the present study had some limitations, Firstly, this study used relatively small sample sizes, thus decreasing statistical power to detect. Secondly, the study is a cross-sectional design and without longitudinal data, which pose explanation mainly focusing on temporary effects, and is difficult to clarify determining causality. Thirdly, Identifying the child trauma is based on the self-reports of patients, some subjects may be willing to recalling negative events, thus recall biases are thought to play a role (43). In consideration of the above-mentioned facts, further research is necessary to make lager scale and long-term investigated research, so as to getting better understand the relationship about the victims of child trauma and suicide in the patients of schizophrenia.

## Conclusion

In conclusion, Child trauma are commonly encountered in SCZ patients, especially, in the relapsed SCZ patients. BMI and emotional neglect may be a specific detective factors for the suicide risk, suicidal ideation and proneness to suicidal behavior in the relapsed SCZ patients. With some of comprehensive understanding in the this filed and that will likely be essential for therapeutic developments and prevention measures.

## Data availability statement

The raw data supporting the conclusions of this article will be made available by the authors, without undue reservation.

## Ethics statement

The studies involving human participants were reviewed and approved by Medical Ethics Committee of the Anhui Mental Health Center. The patients/participants provided their written informed consent to participate in this study.

## Author contributions

CZ and XZ were responsible for study design and manuscript editing. PC was responsible for statistical analyses and manuscript writing. LC, YC, JL, and QX were responsible for literature searches, statistical analyses, and manuscript writing. JG, LZ, FY, XC, and WP were responsible for clinical-scale assessment data collection. All authors contributed to the article and approved the submitted version.

## Funding

This work was funded by Project of Anhui Medical University (2019xkj206), Hospital Project of Hefei Fourth People's Hospital (Grant Number: 2019023), Shanghai Key Laboratory of Psychotic Disorders Open Grant (Grant Number: 13dz2260500), Natural Science Research Projects in Anhui Universities (Grant Number: KJ2020A0218), Applied Medicine Research Project of Hefei Health Committee (Grant Number: Hwk2020zd0016), Applied Medicine Research Project of Anhui Health Committee (Grant Number: AHWJ2021a036), and Project of Central Public Welfare Scientific Research Institutes (Grant Number: GY2020G-3).

## Conflict of interest

The authors declare that the research was conducted in the absence of any commercial or financial relationships that could be construed as a potential conflict of interest. The reviewer YH declared a shared affiliation with the author PJ to the handling editor at the time of review.

## Publisher's note

All claims expressed in this article are solely those of the authors and do not necessarily represent those of their affiliated organizations, or those of the publisher, the editors and the reviewers. Any product that may be evaluated in this article, or claim that may be made by its manufacturer, is not guaranteed or endorsed by the publisher.
